# Editorial: Advancements in smart diagnostics for understanding neurological behaviors and biosensing applications

**DOI:** 10.3389/fncom.2025.1693327

**Published:** 2025-09-16

**Authors:** Saad Arif, Muhammad Zia ur Rehman, Zohaib Mushtaq

**Affiliations:** ^1^Department of Mechanical Engineering, College of Engineering, King Faisal University, Al Ahsa, Saudi Arabia; ^2^Department of Biomedical Engineering, Riphah International University, Islamabad, Pakistan; ^3^Department of Electrical, Electronics and Computer Systems, College of Engineering and Technology, University of Sargodha, Sargodha, Pakistan

**Keywords:** biosensing techniques, smart diagnostics, human-computer interaction, brain-computer interface, intelligent healthcare, physiological monitoring, neurobehavior analysis, machine and deep learning

## Overview

An understanding of neurological behaviors can be well-established by bringing together machine learning (ML) and biosensing techniques. This combination is promising for accelerating smart diagnostics and human-computer interactions (HCI), with the following three aspects necessary to create a real-world impact of this combination. Multimodal and multiscale learning to capture the richness of physiological signals is of primary importance, followed by explainability and clinical acceptability that link model evidence to neurophysiology. Designing the trustworthy pipelines that safeguard privacy and support deployment in clinics, homes, and wearables is of practical significance in this endeavor.

In this pursuit, 10 contributions were submitted to this Research Topic, covering biosensing modalities including electroencephalography (EEG), electrooculography (EOG), electrodermal activity (EDA), functional near-infrared spectroscopy (fNIRS), medical imaging, and electrical impedance tomography (EIT)-based tactile sensing. This Research Topic effectively describes how contemporary learning paradigms such as transformers and state space models, hybrid unsupervised-supervised pipelines, and privacy-preserving training, translate signals into actionable insights while respecting the constraints of clinical and everyday settings.

A foundational theme is the decoding of affective and behavioral states from biosignals for continuous monitoring and neuroadaptive interfaces. Xuanzhi et al. modeled stress from EDA using attention-based sequence learning, showing that temporal context in peripheral signals can support robust continuous assessment, with accuracies reaching above 95% on public datasets. Another study by Usman et al. integrated EEG with eye tracking (ET) to predict real-world choices, illustrating how multimodal fusion and ensemble strategies can extract complementary neural and ocular markers of preference in ecologically valid scenarios. Their approach achieved around 84% accuracy with high precision for positive preferences. Extending behavioral inference to social cognition, Bhutta et al. employed frontal fNIRS in an interactive setting to distinguish deception from truth-telling using deep neural networks, attaining approximately 88–90% accuracy and pointing to the feasibility of decoding complex, spontaneous behaviors beyond controlled paradigms.

Sleep health emerged as a second thematic pillar, benefiting from multimodal, temporally aware modeling across distinct populations. A contribution on sleep staging by Fan et al. transformed EEG and EOG into time-frequency sequences, coupled long-range temporal modeling with multiscale feature extraction, and integrated modalities to mitigate the heterogeneity introduced by obstructive sleep apnea (OSA). This design demonstrates broader applicability beyond healthy cohorts and enhances interpretability for clinical workflows, with performance at approximately 80% in OSA cohorts and improvements over competitive baselines on public datasets. Complementing this system's view, a neonatal study by Siddiqa et al. identified promising electrode configurations and informative signal features that sustain accurate sleep state classification. Notably, a single central channel maintained an accuracy of approximately 81%, and compact left hemisphere montages slightly outperformed right hemisphere channels. The study presented a practical sleep monitoring strategy that prioritizes comfort, safety, and computational efficiency in newborn care.

Methodological innovations in EEG decoding were highlighted by work that blends efficient sequence modeling with targeted attention and multiscale feature design. A study by Li advanced a compact state space architecture paired with pyramidal convolutions and channel-spatial attention to improve EEG classification for brain-computer interfaces (BCI). The findings underscore the potential of latency-conscious designs for real-time use on a standard dataset while achieving approximately 97% performance with strong class-wise balance. In parallel, a clinical study by Umair et al. on major depressive disorder (MDD) detection demonstrated that ensembles leveraging transformer representations can deliver high-accuracy classification from EEG data while operating within a decentralized, split-learning framework that keeps data local across nodes. The approach maintained over 95% accuracy across clients, and reached approximately 99% accuracy in centralized settings, aligning with institutional privacy requirements and offering a viable path to collaborative, ensemble learning without compromising data security.

Beyond electrophysiology, imaging-centric contributions emphasized both diagnostic capability and pipeline security. A large-scale study on karyogram analysis by Tabassum et al. proposed a hybrid approach that pre-trained the proposed classifier on unlabeled images and fine-tuned it to detect structural anomalies, complemented by techniques that localized abnormal regions. This design addresses the pervasive challenge of rarely labeled anomalies and demonstrates near state-of-the-art accuracy (approximately 99%), supporting the early screening of chromosome-related neurodegenerative disorders with neurological impact. Complementing analytics with protection, Asiri et al. developed a lightweight bit-plane encryption scheme for medical images tailored to the internet of things (IoT) and edge devices. By leveraging chaotic map-based shuffling and diffusion, the method achieved high entropy (greater than 7.98), low or negative inter-pixel correlations, a vast key space, and robustness under occlusion. This study presented practical safeguards for data in transit and at rest in resource-constrained environments.

Finally, novel sensing modalities extended the scope of smart diagnostics to child-centered interaction and rehabilitation. Asahi et al. employed EIT-based tactile sensing, which presents a safe, integrated device that classifies children's power vs. precision grips using features derived from voltage patterns and tomographic reconstructions. In a pediatric cohort, accuracies exceeded 85%, illustrating how contact-rich sensing can enable the quantitative monitoring of developing motor skills and inform the design of pediatric HCI.

Several cross-disciplinary and cutting-edge research focuses were explored in this Research Topic. First, multimodal fusion and multiscale representations consistently improve robustness to artifacts and population heterogeneity when EEG, EOG, and ET modalities are hybridized or link time-frequency transforms with attention and dilated convolutions. Second, temporal sequence modeling via transformers and state-space formulations captures long-range dependencies that static models overlook, enabling more reliable decoding of stress, behavior, and sleep dynamics. Third, data-efficiency strategies, including unsupervised pretraining, synthetic data augmentation for class balancing with the synthetic minority over-sampling technique (SMOTE), and targeted feature engineering, addressed unlabeled data and imbalanced class distribution issues, which are common in clinical datasets and rare pathology scenarios. Fourth, interpretability is increasingly embedded through attention mechanisms, multiscale modules, and explicit localization, aligning model outputs with physiological prospects and aiding clinical acceptance. Finally, trustworthy deployment is advanced by privacy-preserving learning that limits data movement, lightweight encryption suited to edge devices, and practical design choices such as electrode optimization and a compact scheme that supports real-time, on-device feasibility.

## Conclusion

The presented contributions validate a promising shift from individual performance gains to cutting-edge integrated pipelines that are multimodal, interpretable, cybersecure, and privacy-preserving by design. They demonstrate that modern sequence models and multiscale representations can decode the complex neurobehavioral characteristics of active and passive brain activities (e.g., stress, consumer choice, deception, and sleep dynamics). It has been shown that data-efficient training enables intricate neuronal signatures to be captured efficiently (e.g., neonatal EEG and rare chromosomal anomalies). The contributions established that privacy-preserving analytics and lightweight cryptography are functional aspects for deployment in clinics and daily life (e.g., split learning sustaining over 95% accuracy, edge encryption with high entropy and resilience). To sustain this momentum, the research community should prioritize prospective and a wide range of demographic validation to establish generalization and to adopt shared data standards and benchmarks to strengthen reproducibility. Collaborative explainability should be adopted with clinicians, patients, and end users to support informed decisions. Decentralized learning and secure edge computing should continue to excel for equitable access. Convergence of ML and biosensing approaches, along with these research commitments, will continue to deliver reliable neurotechnology for diagnosis, monitoring, and HCI across the lifespan.

